# Differential responses of size-fractionated eukaryotic microalgae to ocean alkalinity enhancement in oligotrophic seawaters

**DOI:** 10.1128/aem.00092-26

**Published:** 2026-05-29

**Authors:** Hai-Zhen Bian, Lian-Bao Zhang, Hong-Wei Ren, Ji-Hua Liu, Yu-Bin Hu

**Affiliations:** 1 Institute of Marine Science and Technology, Shandong University 520252, Qingdao, China; 2 Key Laboratory of Land and Sea Ecological Governance and Systematic Regulation, Ministry of Ecology and Environment, Shandong Academy for Environmental Planning 846197 , Jinan, China; 3 Qingdao Key Laboratory Ocean Carbon Sequestration and Negative Emission Technology, Shandong University 520252, Qingdao, China; 4 Shandong Key Laboratory of Intelligent Marine Engineering Geology, Environment and Equipment, Qingdao, China; Colorado School of Mines, Golden, Colorado, USA

**Keywords:** olivine, ocean alkalinization, oligotrophication, eukaryotic microalgae, community assembly processes, size-fraction

## Abstract

Ocean alkalinity enhancement through olivine dissolution represents a critical CO_2_ removal pathway. While laboratory experiments have provided insights into olivine dissolution impacts on eutrophic coastal seawater, ecological responses in offshore oligotrophic seawater remain poorly understood. This study investigated how olivine dissolution affects size-fractionated eukaryotic microalgae through shipboard incubations conducted in the South China Sea under near-*in situ* conditions. Our study demonstrated that olivine addition enhanced the richness and evenness of eukaryotic microalgae, promoted community network stability, and altered the direction of succession in the microbial community. Furthermore, olivine dissolution products modulated the activities of eukaryotic microalgae, which may further influence biological pump efficiency. Beyond the dissolution effects, undissolved olivine particles may also influence microbial community structure by providing attachment substrates and generating localized microenvironments at the particle–water interface. Additionally, olivine addition exerted significantly greater effects on community assembly processes in small-sized (0.2–20 μm) eukaryotic microalgae compared to larger size (>20 µm) fractions.

**IMPORTANCE:**

Models and laboratory experiments suggest that adding the alkaline mineral olivine to seawater is an efficient method for enhancing the ocean’s uptake of atmospheric CO_2_. To further explore the ecological changes following olivine addition, we conducted a shipboard incubation experiment in the oligotrophic waters of the South China Sea. It was found that the dissolution of olivine in seawater affects the activity, community diversity, and stability of eukaryotic microalgae. Additionally, this study reveals that olivine dissolution has a greater impact on smaller-sized eukaryotic microalgae. These findings provide important understandings for the impact of the implementation of olivine-based ocean alkalinity enhancement on eukaryotic microalgae in the oligotrophic waters.

## INTRODUCTION

Since the Industrial Revolution, the massive burning of fossil fuels globally has been emitting substantial amounts of carbon dioxide (CO_2_) into the atmosphere (1–3). Atmospheric CO_2_ concentrations have surpassed 420 ppm (1, 4), resulting in the continued exacerbation of environmental issues, such as global warming and ocean acidification (5, 6). There is broad international consensus that solely reducing CO_2_ emissions is now insufficient to meet the targets set by the 2015 Paris Climate Agreement (7), necessitating enhanced anthropogenic removal of atmospheric CO_2_ (8, 9). The ocean, as the Earth’s largest natural carbon pool (10), absorbs approximately 30% of anthropogenic CO_2_ (11). In recent years, multiple marine-based carbon dioxide removal (CDR) approaches have been proposed, among which ocean alkalinity enhancement (OAE) has been identified as a high-potential technology (12).

OAE, a critical negative emission technology for climate change mitigation, operates by adding alkaline minerals to seawater to achieve enhanced alkalinity (13–15). Olivine exhibits a relatively rapid weathering rate and strong CO_2_ sequestration capacity (16). Its dissolution in seawater not only enhances alkalinity to promote oceanic uptake of atmospheric CO_2_ but also partially mitigates ocean acidification (17, 18), thereby protecting acidification-sensitive biological communities like coral reefs. These mechanisms have been rigorously validated through geochemical modeling simulations and controlled laboratory incubation experiments (13, 19–21). However, the ecological impacts of olivine addition, particularly on eukaryotic microalgae, remain inadequately understood, leading to uncertainties when extrapolating these findings to practical OAE application scenarios.

Phytoplankton are present in all oceans (22), where small pigmented eukaryotes, constituting approximately 5% of phytoplankton cell abundance, account for about one-third of total primary production (23). Consequently, increasing attention has been directed toward the effects of alkaline mineral addition on phytoplankton communities. Most existing studies have focused on olivine incubations conducted in coastal or relatively nutrient-rich waters (24–27). Nevertheless, mechanistic studies examining the responses of size-fractionated eukaryotic microalgal communities to olivine addition in oligotrophic seawater remain relatively scarce. As an alkaline silicate mineral, olivine dissolution in seawater can significantly increase concentrations of total alkalinity (TA), dissolved inorganic carbon (DIC), and dissolved silica (DSi) (16, 19, 28). It is hypothesized that this dissolution could stimulate the growth of phytoplankton like diatoms and increase the efficiency of the ocean’s biological pump (16), thereby enhancing atmospheric CO_2_ drawdown (29). As core drivers of marine primary productivity and ocean carbon sequestration, the response of eukaryotic microalgal activities to olivine addition requires in-depth investigation.

In this study, we conducted a shipboard olivine incubation experiment in the South China Sea during the late autumn to investigate the response of size-fractionated eukaryotic microalgal communities to the olivine dissolution under near-*in situ* conditions in oligotrophic waters. This study aims to assess the ecological impact of olivine as a CDR method in oligotrophic waters.

## MATERIALS AND METHODS

### Experiment setup

The olivine addition incubation experiment was conducted onboard the research vessel “Zhangqian” from 13 to 23 November 2023 (late autumn). Surface seawater (1 m depth, 18.3°C, salinity 33.7, sampling location 114.29°E, 21.94°N [Fig. S1]) was collected using a submersible pump and evenly distributed into nine pre-cleaned 20 L polycarbonate buckets (Nalgene, USA). These were placed under the natural sunlight condition, and the water temperature of the incubation system was controlled by pumping in flow-through *in situ* surface seawater to simulate ambient environmental conditions. After seawater distribution, it was left overnight. Experiments commenced the following day, designated as day 0. A total of nine 20 L incubation buckets were established for the experiment. Three buckets were assigned to the control group (Control), and three to the olivine treatment group (Olivine) for the subsequent 10-day incubation. The remaining three buckets were used for initial background sampling on day 0. No olivine was added to the control group, whereas the treatment group received 1‰ (mass fraction, wt/wt) olivine powder on day 0, following the collection of initial background samples. On days 1, 2, 3, 5, 7, and 9, samples were collected from both the control and the treatment groups. These included the carbonate system (pH, TA, and DIC), calcium ions (Ca^2+^), nutrients, chlorophyll a (Chl-*a*), and dissolved organic carbon (DOC). Microbial communities were collected on days 0, 3, and 9. The olivine powder used in this experiment originated from Yucheng Refractories Co., Ltd. (Xi Xia County, Henan Province, China) and is classified as forsteritic olivine. The particle size of the olivine was measured using a Mastersizer 3000 (Malvern, UK), with size parameters of D_10_ = 6.93 µm, D_50_ = 35.3 µm, and D_90_ = 92.9 µm (“D” refers to particle size distribution. Specifically, D_10_ indicates that 10% of particles have diameters below this value.).

### Environmental parameters collection and analysis

During sampling, seawater was thoroughly mixed to ensure the mass ratio of olivine to seawater remained consistent. Seawater was then drawn using a 250 mL syringe and filtered under positive pressure through a 0.45 μm membrane filter (Millipore, USA). Samples for pH were collected in 40 mL amber borosilicate glass vials and measured on the same day using a pH meter (Star A211, Thermo Fisher Scientific, USA) equipped with a high-precision electrode (8157BNUMD, Thermo Fisher Scientific, USA). The pH electrode was calibrated using three pHNBS buffers (4.01, 7.00, and 10.01, Thermo Fisher, USA), followed by calibration against equimolar Tris buffer (Dickson lab). The resulting pH values were reported on the total hydrogen ion concentration scale at a temperature of 25°C (pH_T25_) with an accuracy better than ±0.003 (30). DIC samples were collected in 125 mL borosilicate glass bottles (CORNING, USA), preserved with 25 μL of saturated mercuric chloride solution, and sealed with a ground-glass stopper. Samples were stored at ambient temperature and later analyzed in the laboratory using an infrared gas analyzer AS-C5 (Apollo SciTech, USA). TA, S, and Ca samples were collected in 125 mL HDPE bottles (Nalgene, Thermo Fisher Scientific, USA). TA was measured via automated potentiometric titration (T960E, Hanon, China) using the Gran method (31). Certified reference material (CRM, Batch 205, Dickson lab, USA) was used for quality control in both TA and DIC measurements (precision: ±2 µmol kg^−1^). Ca samples were analyzed using an automated potentiometric titrator (T960E, Hanon, China) equipped with a Ca^2+^ selective electrode (7321Ca, Hanon). Seawater partial pressure of carbon dioxide (*p*CO_2_) and aragonite saturation (Ω_Ar_) were calculated from TA and DIC data at a temperature of 25°C using the CO2SYS program (Version 2.1) software (32). Nutrient samples were collected in 15 mL HDPE centrifuge tubes (Falcon, Corning Science Mexico S.A. de C.V.) and immediately stored at –20°C; they were thawed to room temperature and analyzed using a continuous flow analyzer AA3 (Auto-Analyzer 3, Seal, Germany). For chlorophyll a (Chl-*a*), 300 mL of seawater was pre-filtered through a decarbonated GF/F filter (47 mm, Whatman, USA). The filtrate was collected in 40 mL amber borosilicate glass vials (Nalgene, Thermo Fisher Scientific, USA) for dissolved organic carbon (DOC) determination. Both the GF/F filters and DOC samples were frozen at –20°C. Back in the laboratory, GF/F filters containing Chl-*a* were extracted in the dark with 5 mL of 90% acetone at 4°C overnight, and Chl-*a* concentration was determined fluorometrically using a Cary Eclipse spectrofluorometer (Agilent Technologies, Santa Clara, CA). DOC concentration was measured using a total organic carbon analyzer (TOC-L CPH, Shimadzu, Japan).

### DNA extraction and sequencing

Under filtration pressure <0.01 MPa, 4 L water samples were sequentially filtered through polycarbonate membranes (47 mm, Millipore, USA) with pore sizes of 20 μm and 0.2 μm. Eukaryotic microalgae collected on the 20 μm pore-size membrane were defined as the large size fraction (>20 µm), while those further collected on the 0.2 μm pore-size membrane were defined as the small size fraction (0.2–20 μm). Prior to analysis, membranes were stored at –80°C in 2 mL sterile tubes. DNA was extracted using the FastDNA Spin Kit for Soil (MP Biomedicals, USA) according to the manufacturer’s protocol. The eukaryotic microalgal V4 region of 18S rRNA genes was amplified using primers 528F (5′-GCGGTAATTCCAGCTCCAA-3′) and 706R (5′-AATCCRAGAATTTCACCTCT-3′). Sequencing was performed on the Illumina HiSeq 2500 platform (Novogene Bioinformatics Technology Co., Ltd., Tianjin, China). All sequencing data generated in this study are deposited in the NCBI database; the accession number for the amplicon data is PRJNA1296817.

### Statistical analyses

Differences in physicochemical parameters between control and treatment groups were assessed using a *t*-test in GraphPad Prism (v 8.4.3). Raw gene sequences obtained from sequencing were denoised using QIIME2 (33), after which high-quality, non-chimeric sequences were clustered into amplicon sequence variants (ASVs) using the DADA2 algorithm (34). Representative ASV sequences were taxonomically annotated against the SILVA database (v138). Following the removal of ASVs annotated as protists, fungi, or unclassified, samples were rarefied to an even sequencing depth of 4,246 sequences (the minimum read count in the matrix). Alpha diversity analysis, Principal Coordinates Analysis (PCoA), Canonical Correspondence Analysis (CCA), and Variation Partitioning Analysis (VPA) were performed using the “vegan” package in R. Alpha diversity metrics assessed eukaryotic microalgal community richness and evenness; PCoA based on Bray–Curtis distances explored β-diversity differences; CCA and VPA analyzed correlations between eukaryotic microalgal communities and environmental factors. Environmental variables for CCA were screened using variance inflation factors (VIF <10), and only axes explaining significant variance (assessed by Monte Carlo permutation test with 999 permutations) are presented in figures. Maximal Information Coefficient (MIC) analysis was conducted using the “Minerva” package; relationships were considered significant and valid if they met MIC ≥ 0.4 and statistical significance (*P* < 0.05) using precomputed *P*-values for species-environment factor associations (35). Co-occurrence networks were constructed using the “microeco” package to investigate changes in the eukaryotic microalgal community after olivine addition. ASVs with relative abundances <0.1% were excluded, and only significant correlations (Spearman’s correlation coefficient |r| ≥ 0.7, *P* < 0.05) were retained. Network structure was visualized using Gephi v0.10.1, and topological properties were calculated using the R package “igraph.”

iCAMP (infer Community Assembly Mechanisms by Phylogenetic-bin-based null model analysis), a phylogenetic bin-based null model analysis method, quantifies the relative contributions of different ecological processes (homogeneous selection, heterogeneous selection, dispersal limitation, homogenizing dispersal, and drift) governing microbial community assembly. This is achieved through two key metrics: the within-bin beta Net Relatedness Index (βNRI) and the modified Raup-Crick metric (RC) (36). The impact of ecological processes at the eukaryotic microalgal community level was quantified by calculating the relative abundance of bins, identifying the dominant bins governed by each process. The statistics were performed in the R package “iCAMP.”

## RESULTS

### Changes in the carbonate system and DSi parameters

**Fig 1:**
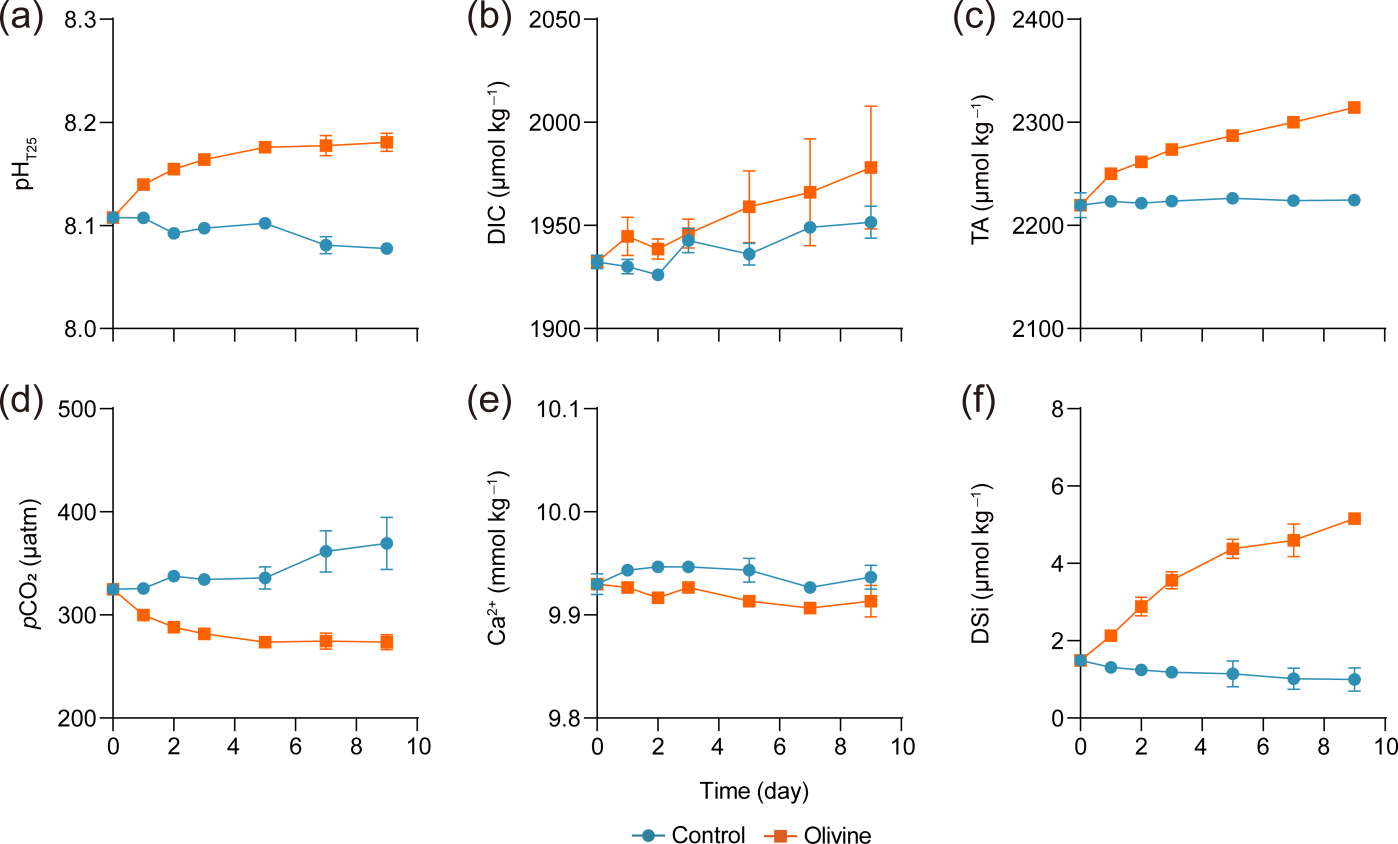
Temporal trends in (**a**) pH_T25_, (**b**) DIC, (**c**) TA, (**d**) *p*CO_2_, (**e**) Ca^2+^, and (**f**) DSi for the control group and the olivine addition group during the shipboard incubation experiment. The vertical bars indicate the standard deviation (*N* = 3).

Over the 10-day experiment, the pH_T25_ in the treatment group showed a continuous increase, reaching 0.073 ± 0.005 units higher than the initial level by day 9. In contrast, the pH_T25_ in the control group showed an overall declining trend, decreasing by 0.032 ± 0.003 compared with the initial value by the end of the experiment. Overall, the pH_T25_ in the treatment group was consistently and significantly higher than in the control group (*P* < 0.05). DIC and TA accumulated continuously in the treatment group, increasing by 46 ± 8 µmol kg^−1^ and 95 ± 4 µmol kg^−1^, respectively. Neither parameter showed significant changes in the control group, and both were significantly lower than in the treatment group (*P* < 0.05). The *p*CO_2_ in the treatment group was significantly lower than in the control group, with the former decreasing slowly during the experiment (51 ± 6 µatm), while the latter increased by 44 ± 21 µatm. These trends in the carbonate equilibrium system indicate enhanced carbon sink capacity due to olivine addition. Ca^2+^ concentration was slightly lower in the treatment group compared to the control over the experimental period. The Ω_Ar_ in the control group remained relatively stable but decreased continuously by 0.20 ± 0.06 over the last 4 days (day 5 onwards). Conversely, the treatment group exhibited a continuous increase of 0.66 ± 0.02 in Ω_Ar_ until the end of the experiment (Fig. 1a through e; Fig. S2a). Notably, dissolved inorganic nitrogen and dissolved inorganic phosphate were below detection limits throughout the experimental period (Fig. S1a through c). In contrast, DSi showed a continuous increase in the treatment group (3.66 ± 0.14 µmol kg^−1^) and a gradual decline in the control group (0.50 ± 0.18 µmol kg^−1^) (Fig. 1f).

### Changes in eukaryotic microalgal community composition and environmental drivers

**Fig 2:**
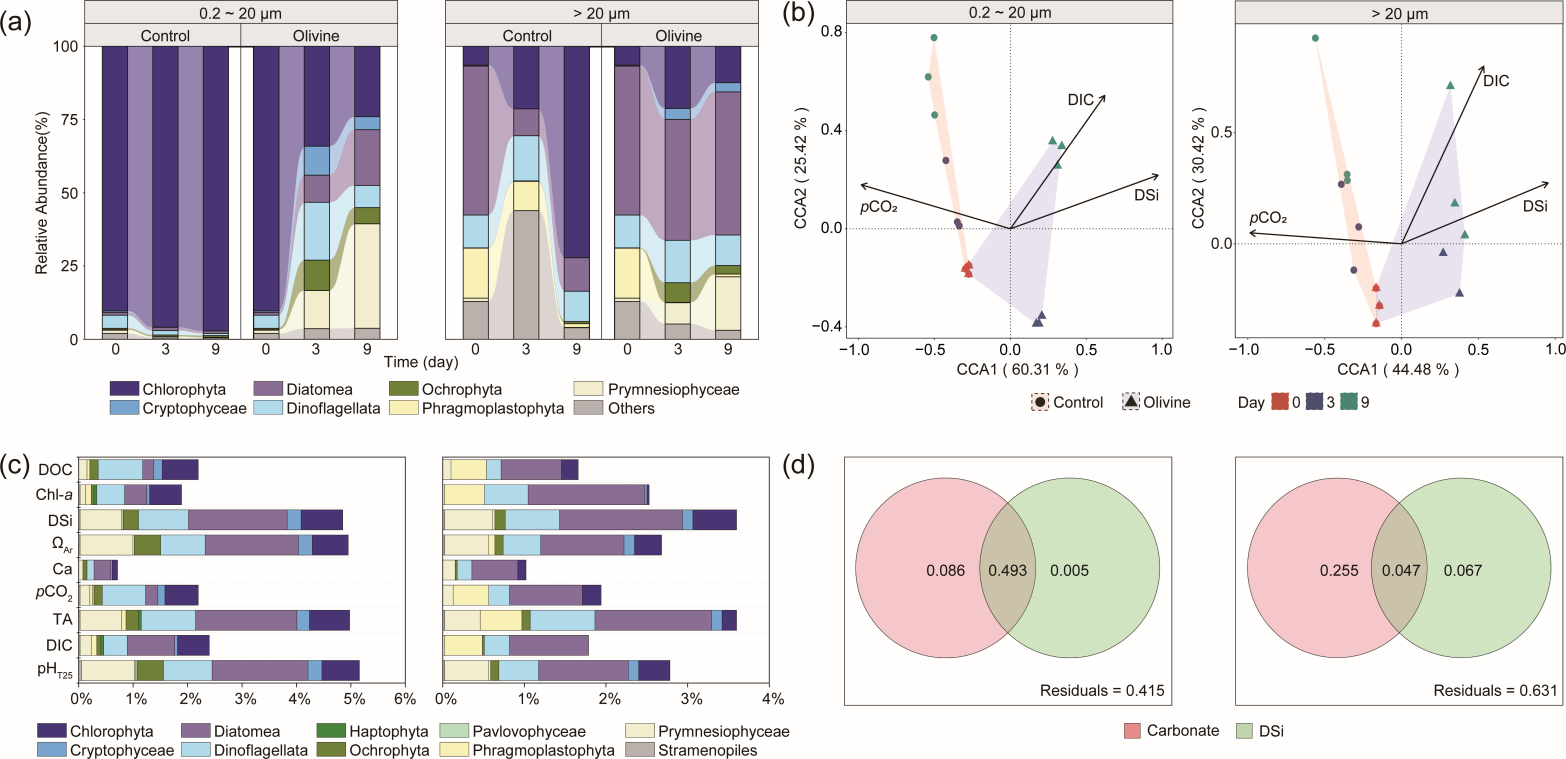
(**a**) Average relative abundance (*N* = 3) of eukaryotic microalgal communities in different size fractions at the phylum level in seawater from the control group and the olivine addition group during 10-day incubation. (**b**) CCA reveals the driving effects of environmental factors (*p*CO_2_, DIC, and DSi) on microbial community grouping (Control and Olivine group). (**c**) MIC analysis showing the contribution of environmental factors (DOC, Chl-*a*, DSi, Ω_Ar_, Ca, *p*CO_2_, TA, DIC, and pH_T25_) to eukaryotic microalgae at the phylum level. (**d**) VPA quantifying the contributions of the carbonate system (pH_T25_, DIC, TA, *p*CO_2_, and Ω_Ar_) and DSi to eukaryotic microalgal communities.

The species composition of experimental samples for the small size fraction (0.2–20 µm) and large size fraction (>20 µm) is shown in Fig. 2a. This study displays the top seven taxa at the phylum level and the top twelve taxa at the class level (with an average relative abundance ≥1% in at least five samples). The results demonstrate a clear response of the microbial community structure to olivine addition. At the phylum level, Chlorophyta consistently dominated the small size fraction in the control group (relative abundance >89.4% throughout the experiment) and exhibited an increasing trend over time. In the treatment group, although Chlorophyta remained the dominant phylum, its relative abundance showed a declining trend. In the large size fraction of the control group, the initial proportion of Chlorophyta was lower than in the small size fraction group, but demonstrated an overall increasing trend during the experiment. Conversely, in the large size fraction of the treatment group, its proportion increased during the mid-experimental phase but decreased in the late phase. Furthermore, in control groups of both particle sizes, the relative abundances of Diatomea and Prymnesiophyceae exhibited declining trends during the experiment, whereas their relative abundances increased significantly in the treatment groups (Fig. 2a). At the class level (Fig. S2), *Mamiellophyceae* (within Chlorophyta) displayed a contrasting dynamic to the overall trend observed at the phylum level, with its relative abundance increasing over time in the treatment group. The dynamics of the remaining classes aligned with those of their corresponding phyla.

CCA evaluated relationships between environmental parameters and eukaryotic microalgal communities, using parameters with variance inflation factors (VIF) <10 for analysis. CCA indicated that *p*CO_2_, DIC, and DSi significantly determine size-fractionated eukaryotic microalgae (Fig. 2b). MIC was employed to detect correlations between individual taxa and multiple environmental parameters (Fig. 2c). For the small size fraction, the highest proportion of taxa showed significant association with pH_T25_ (5.16%). For the large size fraction, the highest proportions were associated with TA and DSi (both 3.60%). All these parameters are directly linked to the olivine addition. Additionally, for the small fraction, Chlorophyta, Diatomea, Dinoflagellata, and Prymnesiophyceae exhibited a relatively high degree of association with all environmental factors. For the large fraction, Diatomea showed higher association proportions across all parameters compared to other taxa. Furthermore, VPA was employed to quantify the influence of environmental factors—comprising the carbonate system (pH_T25_, TA, DIC, *p*CO_2_, and Ω_Ar_) and the nutrient system (DSi)—on microbial community diversity. Environmental factors explained a higher proportion of community variation for the small fraction (58.5%) compared to the large fraction (36.9%). The carbonate system alone contributed the most for both fractions (8.6% and 25.5%, respectively). The shared effect of both environmental factors was substantial for the small fraction (49.3%) but minimal for the large fraction (4.7%) (Fig. 2d). Collectively, MIC, CCA, and VPA analyses demonstrate that the carbonate system and DSi, which were modified by olivine dissolution, play a pivotal role in structuring eukaryotic microalgal communities.

### Analysis of diversity and co-occurrence networks in eukaryotic microalgae

**Fig 3:**
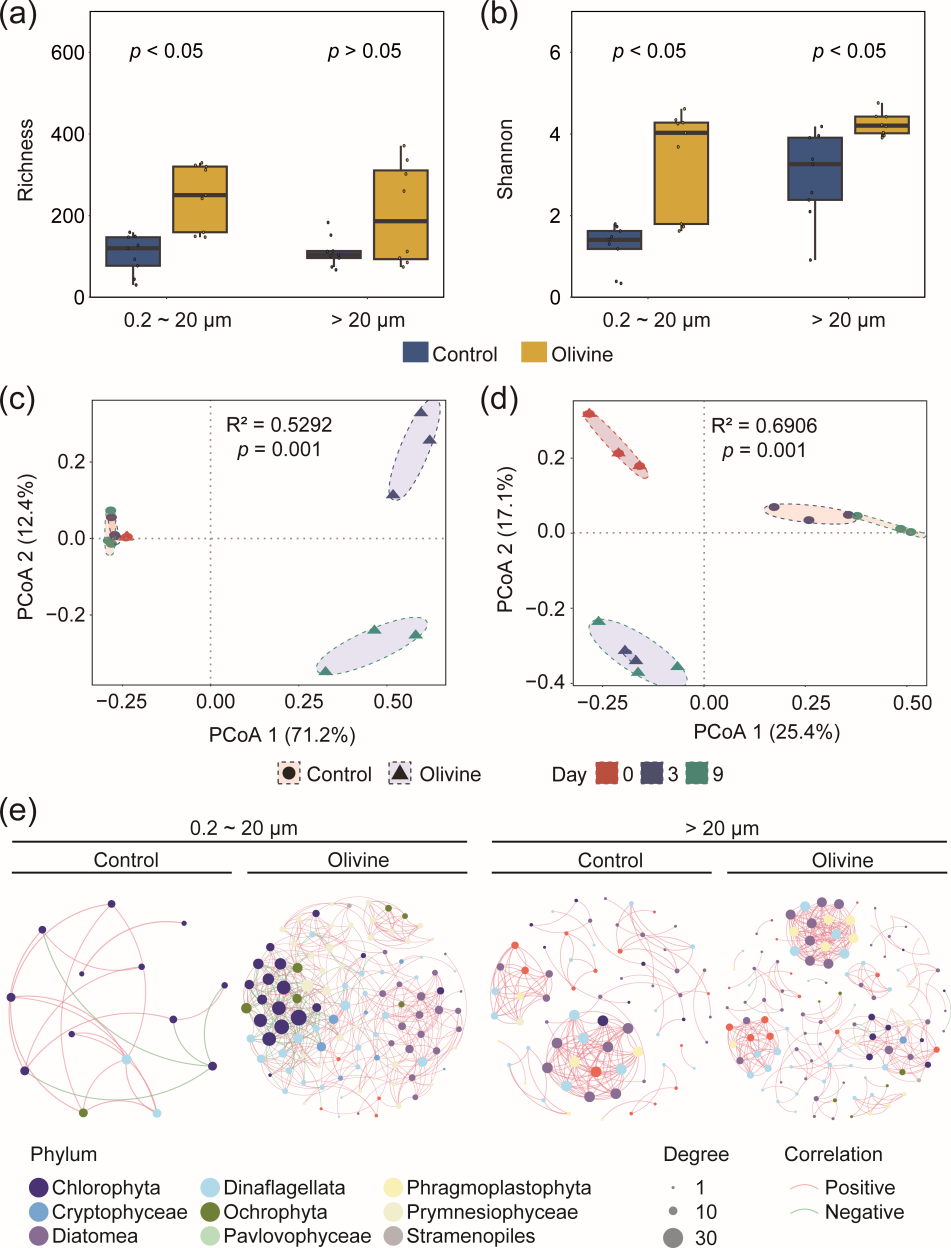
Effects of olivine addition on the α-diversity and community structure of size-fractionated eukaryotic microalgae. Variations in α-diversity (**a: **Richness and **b: **Shannon indices) of size-fractionated eukaryotic microalgal communities between the control and the olivine addition groups. The vertical bars indicate the standard deviation (*N* = 3). PCoA of eukaryotic microalgal communities for the different size fractions in the control group and the olivine addition group. (**c: **Small size fraction [0.2–20 µm] and **d**: large size fraction [>20 µm]). (**e**) Co-occurrence patterns among eukaryotic microalgae across size fractions in the control and olivine addition groups.

Variations in species richness and evenness of microbial communities across different size fractions were systematically assessed using boxplots of α-diversity indices (Fig. 3a and b). The data indicate that for the small size fraction, all α-diversity indices were significantly higher in the treatment group than in the control group. For the large size fraction, only the Shannon and Pielou indices were significantly higher in the treatment group compared to the control; although the other indices were also higher in the treatment group, the differences were not statistically significant (*P* > 0.05). PCoA results indicated that for the small size fraction, the eukaryotic microalgal community structure in the control group did not undergo significant changes over the incubation period. However, the successional trajectory of the community was altered in the treatment group (Fig. 3c). For the large size fraction, the community structure changed over time in both the control and treatment groups, but olivine addition significantly altered its successional trajectory (*P* = 0.001) (Fig. 3d).

By constructing co-occurrence networks of eukaryotic microalgal communities, the impact of olivine addition on microbial interaction patterns across different size fractions was revealed. The small size fraction in the treatment group (114 nodes, 484 edges, and average degree 8.64) formed a significantly more complex network structure compared to the control group (14 nodes, 21 edges, and average degree 3.00), accompanied by a decrease in the proportion of positive correlation edges (control vs. treatment: 80.1% vs. 75%). A similar trend was observed for the large size fraction: olivine addition expanded the network scale (control: 71 nodes, 176 edges, and average degree 4.96; treatment: 128 nodes, 347 edges, and average degree 5.42), and decreased the proportion of positive correlations (100% vs. 97.1%) (Table S1; Fig. 3e). These findings indicate that olivine addition fostered the formation of more complex and tightly connected interaction networks within the microbial communities.

### The importance of different assembly processes

**Fig 4:**
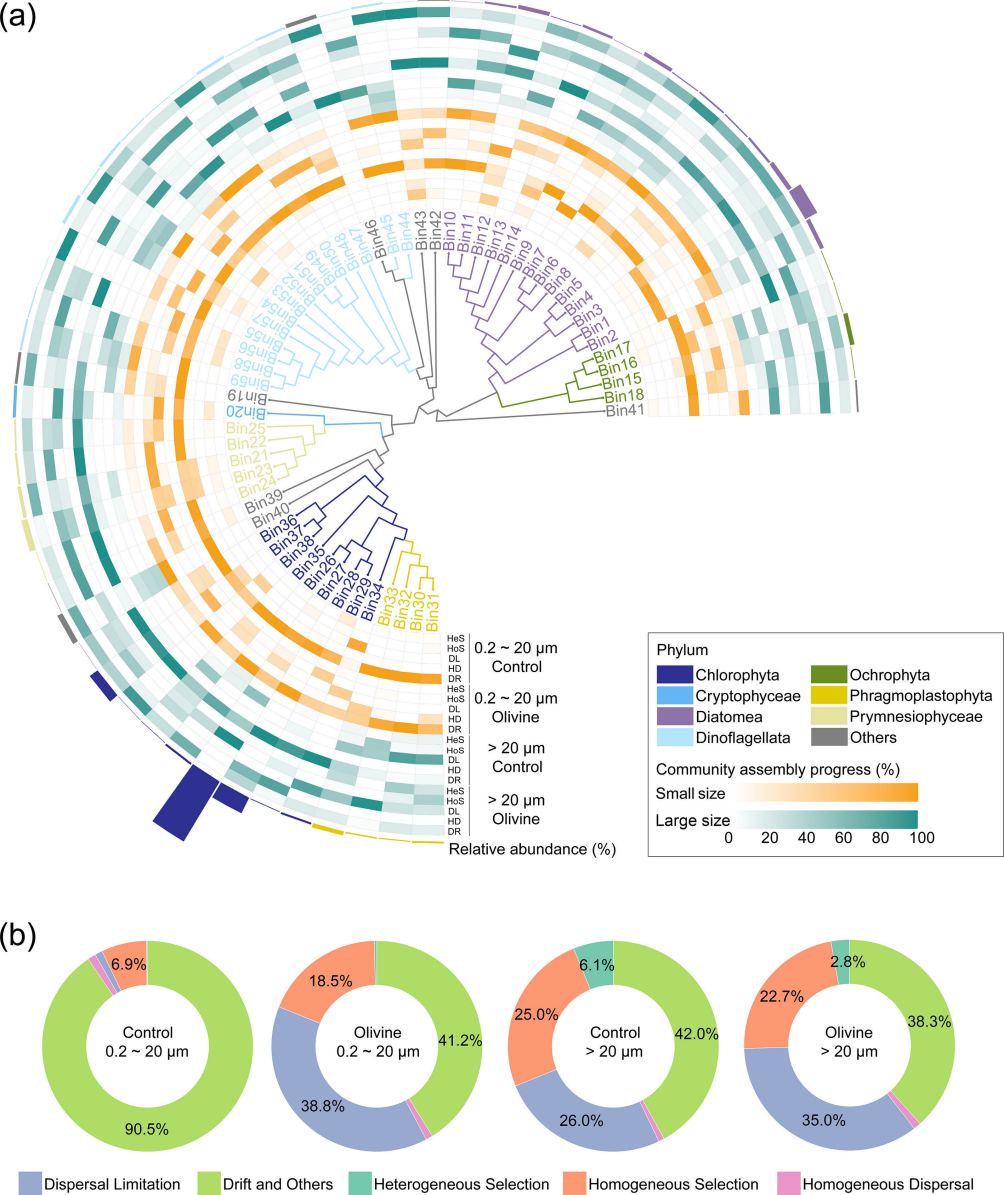
Variations in community assembly processes across different phylogenetic groups. (**a**) Phylogenetic tree, relative importance of different processes, and relative abundance of phylogenetic bins. (**b**) Relative influence of different ecological processes on community assembly.

iCAMP was used to estimate the relative importance of different assembly processes governing community assembly. Assembly processes were categorized into deterministic (homogeneous selection [HoS], heterogeneous selection [HeS]) and stochastic (dispersal limitation [DL], homogenizing dispersal [HD], and drift [DR]) processes. Stochastic processes dominated across all four experimental groups (57%–94%). For the small size fraction in the control group, DR was the dominant process (90.5%). Although the relative importance of DR decreased, it remained the primary process for the small size fraction in the treatment group (41.2%). Olivine addition increased the influence of HoS (from 5.9% to 18.5%) and DL (from 1.2% to 38.8%). For the large size fraction in the control and treatment groups, DR was dominant, but its contribution was lower in the treatment group (38.3%) than in the control (42.0%). Concurrently, the combined contribution of deterministic processes decreased from 31.1% (the control group) to 25.5% (the olivine group), while the contribution of DL increased from 26.0% (the control group) to 35.0% (the olivine group) (Fig. 4b).

In the iCAMP analysis, observed ASVs were partitioned into 59 phylogenetic bins, and the relative importance of processes was assessed for each bin (Fig. 4a). Among them, Bin27, containing *Trebouxiophyceae* (33.2%), exhibited the highest relative abundance among the bins driving assembly processes, followed by Bin28 (*Clade_VII*, 8.5%) and Bin1 (*Coscinodiscophytina*, 8.4%). For the small size fraction in the control group, the contributions of HeS, HoS, DL, HD, and DR were 0.0%, 3.5%, 1.8%, 8.8%, and 86.0%, respectively; while in the treatment group, these values changed to 1.7%, 19.0%, 20.7%, 0.0%, and 58.6%. For the large size fraction in the control group, the contributions were 3.4%, 8.6%, 27.6%, 0.0%, and 60.4%, changing to 1.7%, 11.9%, 42.4%, 3.4%, and 40.7% in the treatment group. When considering the relative abundance of each bin, the relative contribution of DL, DR, HD, HoS, and HeS for the small size fraction changed from 0.9%, 81.6%, 13.4%, 1.9%, and 0.0% in the control group to 15.6%, 73.5%, 0.0%, 8.9%, and 0.2% in the treatment group, respectively. While for the large size fraction, the contributions changed from 10.6%, 56.5%, 0.0%, 36.3%, and 2.8% in the control group to 24.9%, 26.7%, 8.5%, 39.3%, and 0.5% in the treatment group, respectively. The role of DR in the experiment was driven by the response of bins associated with various taxa, while HoS was primarily dominated by bins assigned to Chlorophyta, Cryptophyceae, Diatomea, and Dinoflagellata. Since Chlorophyta bins accounted for the highest proportion (48.5%), they exerted a decisive influence on driving community assembly processes, followed by Diatomea bins (19.6%).

## DISCUSSION

While prior investigations have assessed the impacts of olivine-based OAE on phytoplankton in the oligotrophic ocean (15, 26, 37, 38), research examining the responses of size-fractionated eukaryotic microalgal communities remains scarce. As vital primary producers in the ocean, eukaryotic microalgae form a fundamental foundation of the marine food web (39, 40), and play a significant role in the marine carbon cycle (41). However, they are also sensitive to environmental changes (42). Our study reveals that olivine addition exhibits a dual regulatory effect on both ocean carbon sink capacity and ecosystem function.

Following olivine addition, *Trebouxiophyceae* and *Clade_VII*, although remaining dominant algal groups, exhibited a declining trend in relative abundance. Conversely, the relative abundance of *Mamiellophyceae* (also within Chlorophyta) increased continuously. Related studies indicate that certain Chlorophyta (e.g., *Trebouxiophyceae*, *Clade_VII*) typically dominate in oligotrophic waters (43–45), whereas *Mamiellophyceae* tend to dominate in nutrient-rich (eutrophic) waters. This suggests divergent metabolic strategies among different Chlorophyta lineages in response to increased silicate concentrations (46). The release of silicate from olivine altered nutrient composition, consequently influencing the Chlorophyta community structure. Research has found that under nitrate-limiting conditions, low pH (high *p*CO_2_) and low DSi concentrations adversely affect diatoms (*Bacillariophyceae*, *Coscinodiscophytina*, and *Mediophyceae*) (47, 48). Olivine addition significantly increased pH and DSi concentration; thereby, the aforementioned diatom groups showed significantly higher relative abundances in the treatment group than the control across all size fractions (Fig. S2). This indicates that olivine addition would promote diatom growth. Furthermore, studies by Li et al. (16) and Zhang et al. (49) found a synergistic effect between olivine dissolution and diatom growth, which may further enhance carbon sequestration efficiency. The increase in the relative abundance of certain *Prymnesiophyceae*, which are calcifying microorganisms capable of carbon fixation (50–52), on the one hand, could enhance the efficiency of the biological carbon pump; on the other hand, this increase may contribute to the lower calcium ion concentration observed in the treatment group compared to the control (Fig. 1b).

The restructuring process of microbial communities was comprehensively elucidated via multidimensional systems biology analyses. The results from CCA, MIC, and VPA demonstrated that seawater carbonate system parameters (pH_T25_, DIC, TA, and *p*CO_2_) and DSi, which were directly influenced by olivine dissolution, profoundly shaped eukaryotic microalgal communities. α-diversity metrics revealed increased richness and evenness among eukaryotic microalgae. The PCoA analysis based on Bray–Curtis distances indicated that olivine addition significantly altered the community structure and the direction of succession in eukaryotic microalgal communities. Microbial co-occurrence network analysis further showed that olivine addition substantially enhanced network complexity while reducing the proportion of positive correlations among microorganisms. This shift may potentially enhance community resistance to disturbance, thereby contributing to enhanced community stability (53). It should be noted that, in addition to changes in the carbonate system and DSi release, the dissolution of olivine may also release trace metals such as Ni and Cr (54, 55). Although studies conducted under comparable experimental conditions have generally suggested that the trace metals released from olivine are unlikely to reach toxic levels (29, 56), their potential ecological effects cannot be completely ruled out in the absence of direct measurements. More studies are needed to evaluate this issue by combining measurements of trace metal concentrations, bioavailability, and toxicity thresholds.

The assembly processes of microbial communities involve both stochastic and deterministic processes, and evaluating the importance of these different processes is one of the core questions in microbial ecology (57). The importance of each process for both size fractions was altered by olivine addition, with more pronounced changes observed for the small size fraction, indicating that small-sized eukaryotic microalgae were more easily affected by olivine. Furthermore, DL primarily influences microbial assembly processes by reducing the capacity for biological exchange between communities (58). This study observed significantly increased proportions of dispersal limitation in the experimental group compared to the control group, with notably more pronounced changes for the small size fraction. In addition to changes in the carbonate system and DSi, olivine particles themselves may exert additional effects on community structure through particle-associated processes. Previous studies have demonstrated that particle-associated communities differ significantly from free-living communities in both composition and function (59–61). Particles not only serve as physical substrates for attachment, but can also generate distinct microenvironments through biofilm formation and interfacial chemical gradients, thereby exerting selective effects on microbial taxa (59, 62, 63). Moreover, restricted mass exchange within the particle boundary layer may alter nutrient diffusion and cell–cell interactions, ultimately influencing community assembly processes (64). Theoretical and experimental studies further suggest that, under continuous mixing conditions, small-sized cells are more likely to enter the particle boundary layer and undergo transient attachment or co-sedimentation, and their higher surface-area-to-volume ratio may enhance their sensitivity to interfacial chemical gradients (64, 65). Cells attached to or temporarily retained on particle surfaces may experience reduced mobility and exchange capacity, thereby strengthening spatial structuring and increasing the contribution of dispersal limitation to community assembly (59, 63, 65). Therefore, the greater sensitivity of the small size fraction to olivine addition observed in this study may result from the combined effects of dissolution products and particle-associated interfacial processes. In addition, enhanced DL typically leads to increased community dissimilarity (58), a relationship validated by our PCoA results. While existing research has not reached a consensus on how environmental stress influences community assembly processes (36, 66), our findings demonstrate that olivine addition increases the contribution of deterministic processes for the small size fraction but has the opposite effect for the large size fraction. This difference may be linked to the higher environmental sensitivity of the small size fraction.

### Conclusion

This study demonstrates that the addition of olivine in low-latitude oligotrophic sea areas differentially impacts eukaryotic microalgal communities across size fractions. Olivine dissolution products influence eukaryotic microalgal activities, potentially enhancing biological carbon pump efficiency. Furthermore, changes in the community structure and assembly processes of size-fractionated eukaryotic microalgae reveal the complex coupled effects of both olivine dissolution products and particles on these communities. It should be noted that, in the open ocean, factors such as hydrodynamic mixing, particle settling, and dilution effects may substantially alter the residence time and dissolution rate of olivine particles, as well as their interactions with microorganisms. Future studies should further evaluate the long-term ecological impacts of olivine-based ocean alkalinity enhancement through investigations that closely approximate realistic deployment scenarios.

## SUPPLEMENTAL MATERIAL

Supplemental material aem.00092-26-s0001.docx Fig. S1 to S4; Table S1.

ASM does not own the copyrights to Supplemental Material that may be linked to, or accessed through, an article. The authors have granted ASM a non-exclusive, world-wide license to publish the Supplemental Material files. Please contact the corresponding author directly for reuse.
