# Supplementary material for: Differential responses of size-fractionated eukaryotic microalgae to ocean alkalinity enhancement in oligotrophic seawaters
Source: Appl Environ Microbiol. 2026 May 29;92(6):e00092-26. doi: 10.1128/aem.00092-26 (PMC13274457; doi:10.1128/aem.00092-26)
Supplement: Supplemental material — Fig. S1 to S4; Table S1. [file aem.00092-26-s0001.docx]

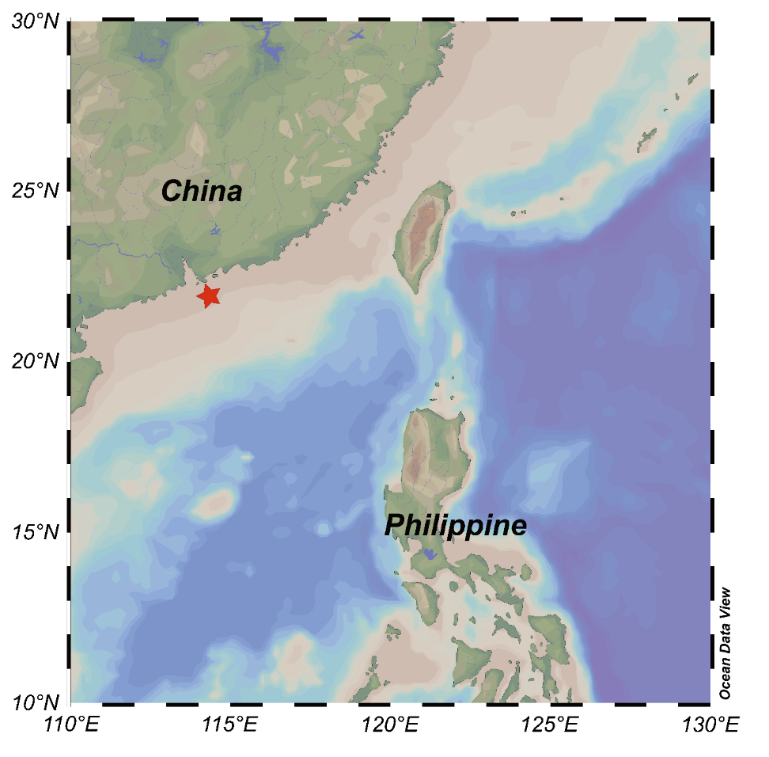
**Supplementary material**

**Figure S1.** Sampling site for incubation experiment in the South China Sea (114.29°E, 21.94°N).


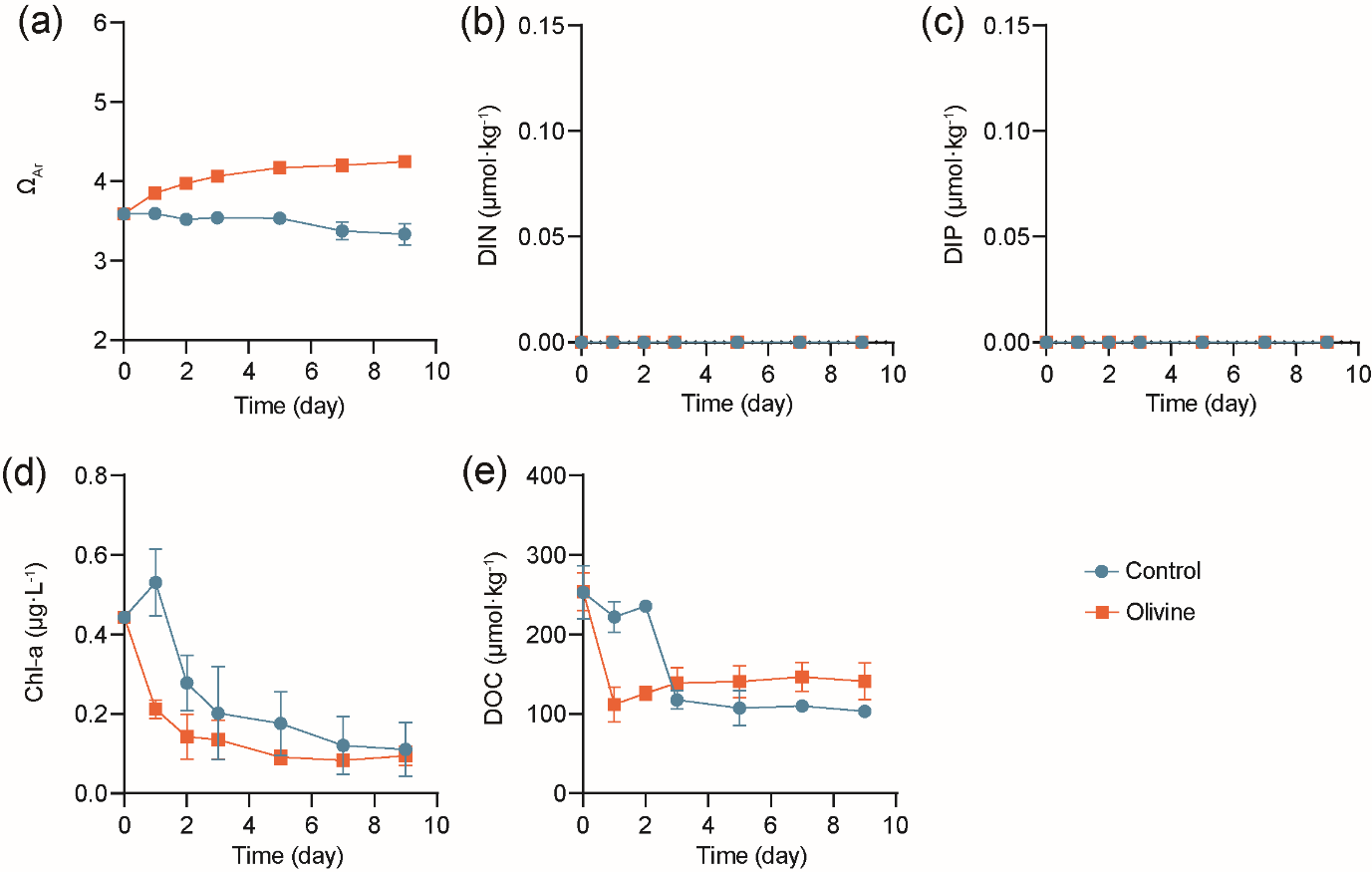


**Figure S2.** Temporal trends in Ω_Ar_ (a), DIN (b), DIP (c) Chl-*a* (d) and DOC (e), for the control group and the olivine addition group during the shipboard incubation experiment. The vertical bars indicate the standard deviation (N = 3).


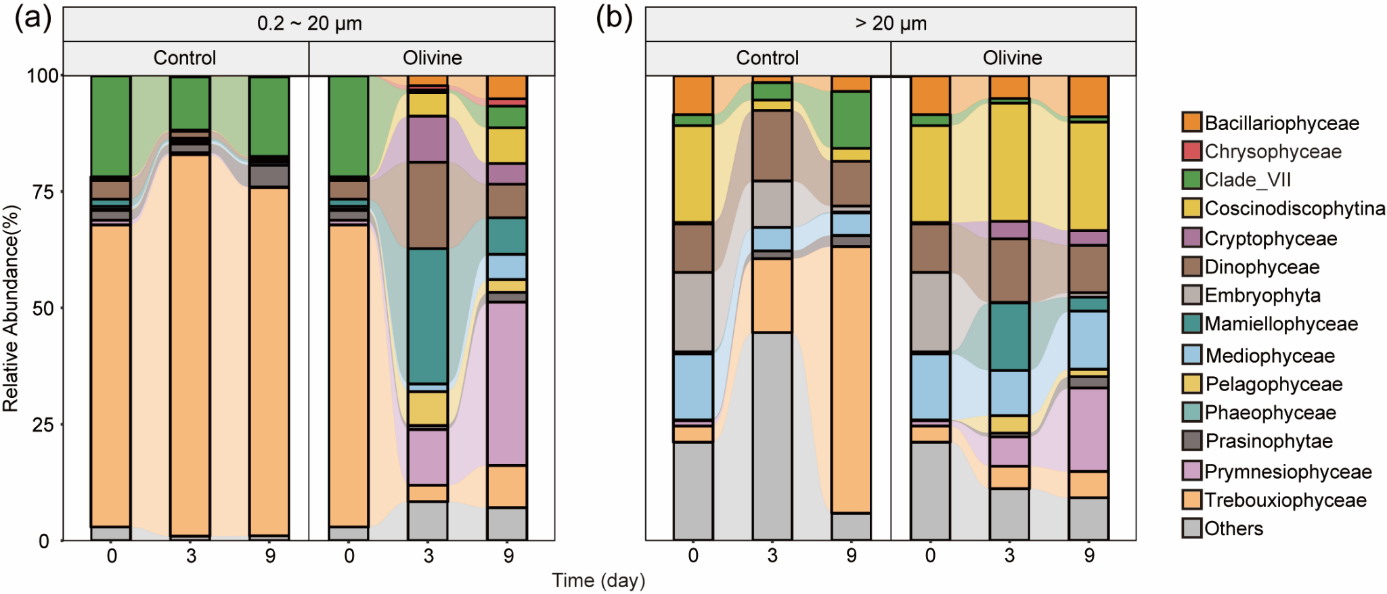


**Figure S3.** Average relative abundance (N = 3) of eukaryotic microalgal communities in different size fractions ((a) 0.2 – 20 µm and (b) > 20 µm) at the class levels in seawater from the control group and the olivine addition group during ten-day incubation.

**
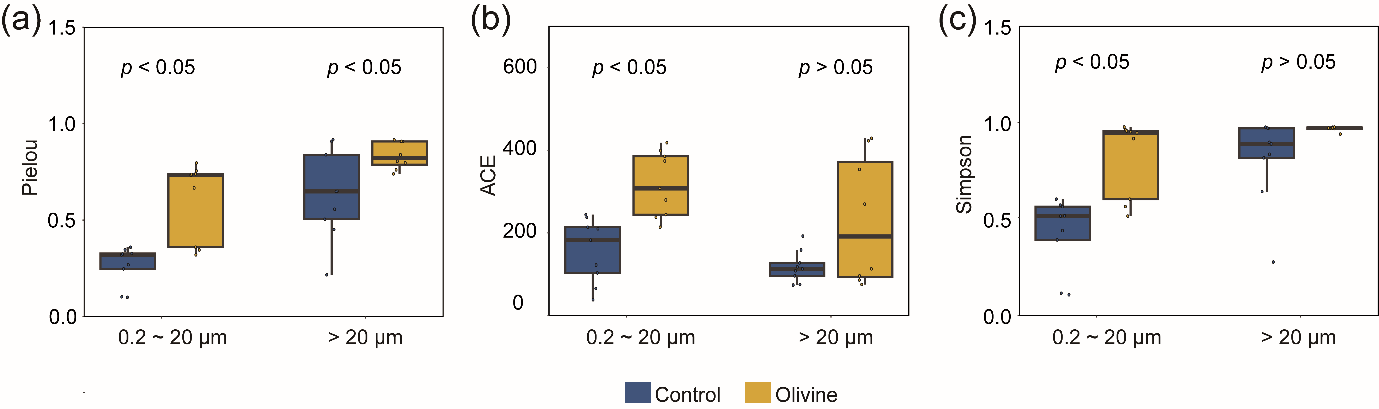
**

Figure S4. Variations in α-diversity ((a) Pielou (b) ACE and (c) Simpson indices​​) of size-fractionated eukaryotic microalgal communities between the control and the olivine addition groups. The vertical bars indicate the standard deviation (N = 3).

**Table S1.** Network Parameters of Eukaryotic Microalgae Across Size Fractions in Control and Olivine Addition Groups.

| **​Filter_pore_size** | **0.2 − 20 µm** | | **> 20 µm** | |
| --- | --- | --- | --- | --- |
| **Properties** | **Control** | **Olivine** | **Control** | **Olivine** |
| Nodes | 14 | 112 | 71 | 128 |
| Edges | 21 | 484 | 176 | 347 |
| Positive edges | 17 | 363 | 176 | 337 |
| Negative edges | 4 | 121 | 0 | 10 |
| Average_degree | 3.00 | 8.64 | 4.96 | 5.42 |
| Average_Weighted_Degree | 2.70 | 7.83 | 4.90 | 5.33 |
| Average_Clustering_Coefficient | 0.77 | 0.53 | 0.90 | 0.78 |
| Average_path_length | 1.20 | 3.51 | 1.15 | 2.87 |
| Network_diameter | 3 | 9 | 4 | 9 |
| Clustering_coefficient | 0.69 | 0.55 | 0.99 | 0.91 |
| Density | 0.23 | 0.08 | 0.07 | 0.04 |
| Heterogeneity | 0.41 | 0.63 | 0.92 | 0.85 |
| Centralization | 0.15 | 0.16 | 0.11 | 0.08 |
| Modularity | 0.54 | 0.56 | 0.68 | 0.79 |
